# Lactate Level Predicts Mortality in Patients with Upper Gastrointestinal Bleeding

**DOI:** 10.1155/2019/5048078

**Published:** 2019-10-24

**Authors:** Muge Gulen, Salim Satar, Adnan Tas, Akkan Avci, Hakan Nazik, Basak Toptas Firat

**Affiliations:** ^1^Adana City Training and Research Hospital, Department of Emergency Medicine, Adana, Turkey; ^2^Adana City Training and Research Hospital, Department of Gastroenterology, Adana, Turkey; ^3^Adana City Training and Research Hospital, Department of Gynecology and Obstetrics, Adana, Turkey

## Abstract

**Background and Objectives:**

The aim of this study was to show whether the level of lactate in venous blood compared with the Glasgow-Blatchford Bleeding Score (GBS), in patients diagnosed with upper gastrointestinal system (UGI) bleeding in the emergency department, will help to predict the need for transfusion and prognosis.

**Materials and Methods:**

Patients with UGI bleeding who were admitted to the emergency department were included in the study. The parameters age, gender, referral complaints, comorbidities, lactate levels in venous blood, GBS, endoscopy findings, length of hospital stay, transfusion amount, and outcome of patients were recorded in the data collection form.

**Results:**

A total of 139 patients were included in the study. The most common complaints were melena (38.1%) and hematemesis (32.4%). The most frequent endoscopic diagnosis was duodenal ulcer (40.3%). The cutoff value of the venous blood lactate level for the prediction of the need for red blood cell transfusion was 1.58 mmol/L, and the cutoff value for GBS was 9.5. While 124 patients were discharged, 15 patients died. The mean value of venous lactate in survived patients was 2.37 mmol/L and 4.80 in dead patients. This difference was statistically significant (*p* = 0.044). The cutoff value of lactate for the prediction of mortality was 2.32 mmol/L, and the cutoff value for GBS was 13.5.

**Conclusions:**

The venous blood lactate value of a patient who was admitted to the emergency department with UGI bleeding might be helpful in predicting the transfusion needs of the patient and predicting the mortality.

## 1. Introduction

Upper gastrointestinal system (UGI) bleeding is one of the most common reasons of admission to the emergency department [1]. Despite all advances in pharmacological and interventional treatment methods, the mortality rate is still between 4 and 14% [2, 3].

Many risk scores have been developed for the early prediction of the high mortality and morbidity rates of UGI bleeding. With the scoring systems developed, whether the patients can be discharged early, the need for blood transfusion, urgent endoscopy and surgical intervention, and the risk of recurrence and mortality are tried to be estimated [4, 5]. The Glasgow-Blatchford Bleeding Score (GBS) system is a scoring system developed to estimate the need for clinical intervention (blood transfusion, endoscopy, or surgery) in patients with UGI bleeding, using basic clinical and laboratory variables, without the use of endoscopic data [6, 7]. However, evidence of prediction of transfusion requirement, recurrent bleeding, and mortality was also shown in studies with GBS [7].

Lactate is a useful prognostic biomarker that can be measured from both venous and arterial blood, showing tissue hypoxia and hypoperfusion. Studies on lactate focused on the relationship between mortality and morbidity, especially in septic patients and intensive care patients. Many medical conditions such as sepsis, seizures, intoxication, severe trauma, hypovolemic, or cardiogenic shock may increase the level of lactate. Serum lactate elevation is also used to estimate the severity of the disease and the risk of mortality [8–10]. Lactate, although elevated in most severity patients admitted to the emergency department with UGI bleeding, is still not routinely involved in any risk classification [11].

The aim of our study was to show whether the level of lactate in venous blood, compared with GBS, in patients presenting with UGI bleeding to the emergency department, will help to predict the need for transfusion, length of hospital stay, and prognosis.

## 2. Materials and Methods

The observational, prospective study was carried out with the joint work of Adana City Training and Research Hospital Adult Emergency Medicine Clinic and Gastroenterology Clinic. Patients with UGI bleeding who were admitted to the emergency department between 1 June 2018 and 31 May 2019 were included in the study. The study was started after the approval of the Cukurova University Medical Faculty Non-Interventional Clinical Research Ethics Committee meeting numbered 77 and Decision 7, dated 4 May 2018, was taken.

### 2.1. Patients

A total of 139 patients over 18 years of age who were successively admitted to the emergency department during the study period with complaints of hematemesis, melena, dizziness, or syncope and with a diagnosis of UGI bleeding with endoscopy were included in the study. Patients with lower gastrointestinal bleeding, who did not accept endoscopy, and who had an infection in addition to UGI bleeding were excluded from the study. Informed consent was collected from the patients or from their next of kin if they were unable to consent.

### 2.2. Data Collection and Measurements

In addition to demographic information such as age and gender, referral complaints, comorbidities, lactate levels in venous blood, Glasgow-Blatchford scores, endoscopy findings, hospitalization duration, transfusion amount, and outcome of patients were recorded in the data collection form.

Blood transfusion decision was planned according to the hemodynamical status and hemoglobin values of the patients. If patients with active bleeding and hypovolemia were not hemodynamically stable with appropriate fluid resuscitation, blood transfusion was performed even if the hemoglobin level was normal. Blood transfusion was started in patients with less than 7 g/dL hemoglobin value even in hemodynamically stable patients. The target hemoglobin level was >9 g/dL in patients with cardiovascular disease and 8 g/dL in patients with portal hypertension.

GBS was calculated according to the vital signs and laboratory parameters of the patients at the time of admission in the emergency room. This score is a risk score based on clinical and laboratory parameters. Scoring is made based on blood urea nitrogen (BUN), hemoglobin value, systolic blood pressure, heart rate, and existence of melena, syncope, congestive heart disease, and liver disease. It can be a minimum of 0 and a maximum of 23. Initial studies have shown that patients with a score of “0” are at very low risk for adverse clinical outcomes and are unlikely to benefit from therapeutic endoscopy. These patients are said to be safely discharged from the emergency department without endoscopy [6].

The venous blood lactate level was measured by Radiometer ABL90 FLEX (Radiometer, Copenhagen, Denmark). Lactate levels were studied from venous blood before any medical treatment at the time of referral to the emergency room.

### 2.3. Primary Endpoint

The effect of calculated GBS and lactate levels on mortality, red blood cell transfusion requirement, and duration of hospitalization were compared. Primary endpoint for this study is mortality during hospitalization. Secondary endpoints include the need for red blood cell transfusion and the number of days of hospitalization.

### 2.4. Statistical Analysis

The SPSS 21 package program was used for the statistical evaluation of the data obtained in the study (SPSS Inc., Chicago, Illinois, USA) [12]. While continuous data were summarized as the mean and standard deviation, categorical data were summarized in terms of numbers and percentages. The Kolmogorov-Smirnov test was used to compare the mean values of the parameters, and for evaluations with a histogram, the Student *t*-test was used in cases where the variables were normally distributed, and the Mann–Whitney U test was used when they were not normally distributed. Pearson correlation analysis was used to explain the relationship between two parametric numerical variables. The receiver operating characteristic (ROC) curve was used to determine the accuracy of clinical scores and lactate levels in measuring mortality. According to this method, the criterion for the best test definition was accepted as 100% sensitivity, false positivity is zero (1‐specificity = 0), the area under the curve (AUC) is 1, and the diagnostic value of AUC is *p* < 0.05. The Youden index, where the highest sensitivity and specificity of the ROC curve is taken, was used to determine the cutoff value. Sensitivity and specificity parameters were calculated with a 95% confidence interval in order to determine the accuracy of the diagnostic test. *p* < 0.05 was taken as the statistical significance level.

## 3. Results

### 3.1. Population Characteristics

**Figure 1:**
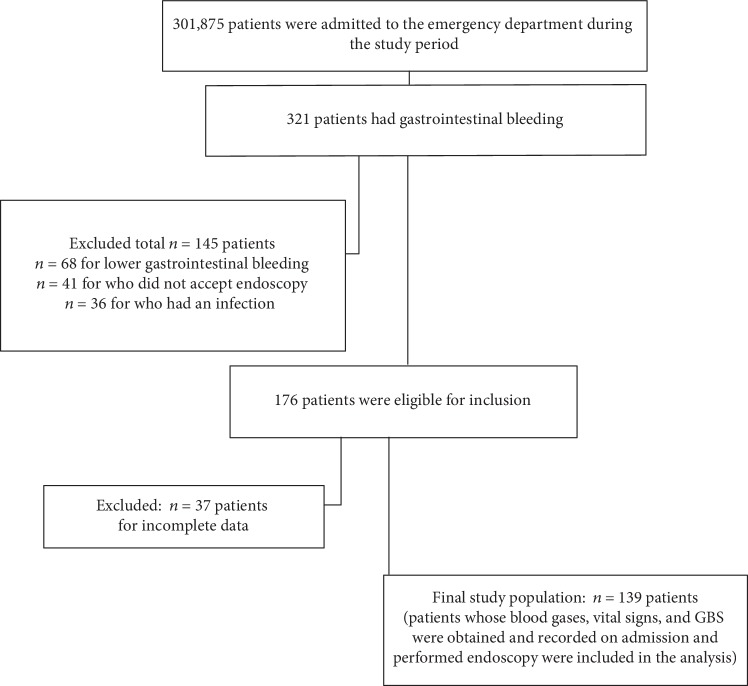
Flow chart of the patients included in the study.

The final study population included 139 patients whose flowchart is given in Figure 1. 28.1% (*n* = 39) of the patients were female, and 71.9% (*n* = 100) were male. The mean age of the patients was 63.34 ± 17.06 years (min 21 years–max 95 years). The most common complaints were melena (38.1%) and hematemesis (32.4%). The most common comorbidity was heart disease (36%). The most common medication that could cause bleeding was antiplatelet (35.3%). The most frequent endoscopic diagnosis was duodenal ulcer (40.3%). 46.04% of the cases had tachycardia (pulse > 100 beats/min), and 21.5% had hypotension (systolic blood pressure < 90 mmHg).

Characteristics of patients according to the requirement of transfusion and survival are summarized in Tables 1 and 2.

### 3.2. Lactate Parameters and GB Scores with Prediction for Outcomes

114 (82%) of the patients had a red blood cell transfusion; 44 (31.6%) had both red blood cell and fresh frozen plasma transfusion. The correlations between red blood cell transfusion and clinical scores showed that the need for transfusion with erythrocyte suspension showed a statistically significant but weak correlation with lactate (*r* = 0.117; *p* = 0.044), and it was statistically significant and moderately correlated with GBS (*r* = 0.520; *p* < 0.001).

**Figure 2:**
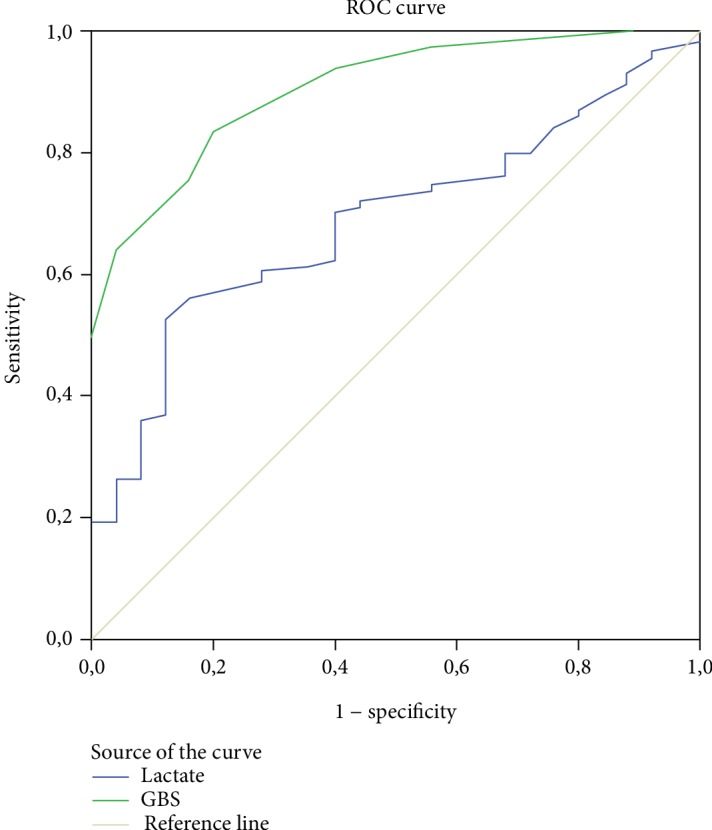
ROC curves showing comparisons of lactate and GBS in predicting need for red blood cell transfusion.

The graph of the ROC analysis to determine the prediction of the need for transfusion of red blood cell by lactate values and GBS is presented in Figure 2. In the conducted analytical evaluations, the AUC value of GBS (AUC: 0.904, 95% CI 0.848-0.961, *p* = <0.001) was found to be higher than that of lactate (AUC:0.689, 95% CI 0.590-0.788, *p* = 0.003). When the cutoff value of lactate for the prediction of the need for red blood cell transfusion was taken as 1.58 mmol/L, the sensitivity was determined as 70.3% and specificity as 60%; when the cutoff value for GBS was taken as 9.5, sensitivity was calculated as 83.3% and specificity as 80% (Table 3).

While 124 (89.21%) of the patients were discharged, 15 (10.79%) were dead. The mean number of days of hospitalization of all patients was 5.22. The mean day of hospitalization for patients who were discharged was 5.47 ± 4.07 (min: 1; max: 24), and 3.2 ± 3.16 (min: 1; max: 13) was the mean day of hospitalization for dead patients. The difference between the duration of hospitalization between discharged and dead patients was found to be statistically significant (*p* = 0.040).

Correlations between hospital stay duration and clinical scores showed that the duration of stay of patients weakly but statistically significantly correlated with GBS (*r* = 0.258; *p* = 0.002); as this score increased, the duration of stay was also seen to increase. However, there was no statistically significant correlation between the lactate level (*r* = 0.137; *p* = 0.114) and the hospitalization duration.

The mean lactate value measured from venous blood was 2.63 mmol/L in all patients at the time of admission. The mean value of venous lactate in nonmortal patients was 2.37 mmol/L and 4.80 in mortal patients. This difference was statistically significant (*p* = 0.044).

The mean GBS of all patients was 11.5. The mean GBS was 11.3 in nonmortal patients and 13.3 in the mortal group. This difference was statistically significant (*p* = 0.040).

**Figure 3:**
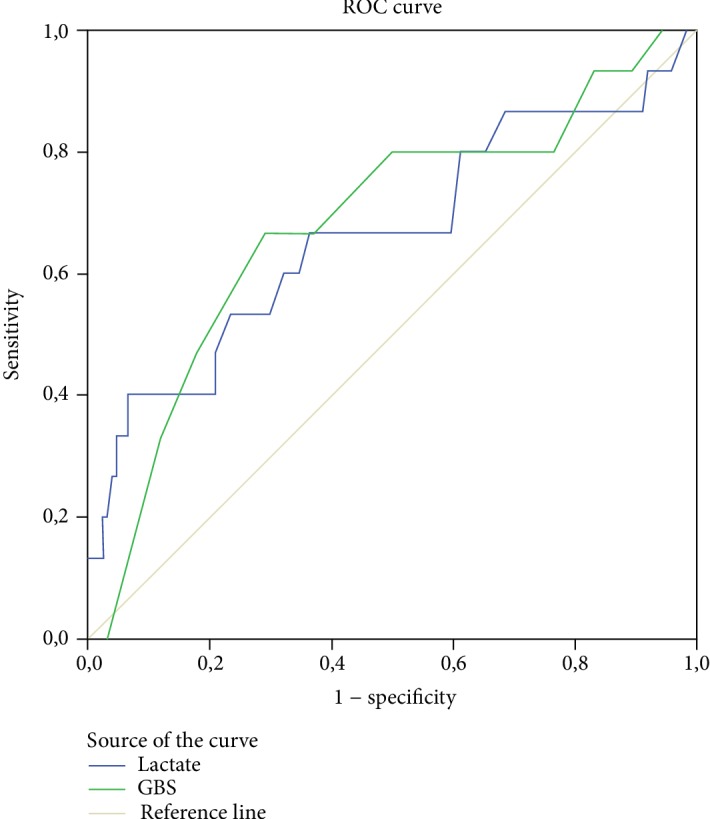
ROC curves showing comparisons of lactate and GBS in predicting hospital mortality.

The graph of the ROC analysis to determine the predictive properties of lactate values and GBS in the patient group is presented in Figure 3. In the conducted analytical evaluations, the AUC value of GBS (AUC: 0.683, 95% CI 0.530-0.835, *p* = 0.021) was found to be higher than the value of lactate (AUC: 0.664, 95% CI 0.495-0.834, *p* = 0.038). When the cutoff value of lactate for the prediction of mortality was taken as 2.32 mmol/L, the sensitivity was determined as 66.7% and specificity as 63.7%; when the cutoff value for GBS was taken as 13.5, sensitivity was calculated as 66.7% and specificity as 71% (Table 4).

## 4. Discussion

Many risk scores have been developed for the early prediction of the high mortality and morbidity rates of UGI bleeding. The objective of the developed scoring systems is to determine low-risk patients early and discharge them and to enable the early access of high-risk patients to the emergency critical care that they need [13]. The most important outcome for these risk scores is the mortality of patients. Other important outcomes are patients' need for blood transfusion, emergency endoscopy and surgical intervention, risk of relapsing, and length of hospital stay [4]. In our study, GBS and lactate were compared in terms of their ability to predict the need for transfusion, number of days of hospitalization, and mortality in patients who are admitted with UGI bleeding. Our study is important because, unlike other studies, the relationship between the serum lactate level and red blood cell transfusion requirement and mortality is similar to that of GBS. We found that if the serum lactate level was higher than 1.58 mmol/L, the need for erythrocyte suspension increased, and the mortality is increased significantly if it was more than 2.32 mmol/L.

Hyperlactatemia can be seen during many conditions that cause tissue hypoxia and organ dysfunction, such as shock, sepsis, seizure, and serious trauma. In anaerobic conditions, pyruvate is converted to lactic acid by lactate dehydrogenase. Hyperlactatemia seen during UGI bleeding occurs through many different mechanisms. The tissue hypoxia state caused by hypovolemia due to hemorrhage causes more lactate production than can be used due to the anaerobic conditions it creates, and hyperlactatemia is developed [14]. In addition, experimental studies have shown that the intestine is sensitive to hypoperfusion. While there is no change in systemic oxygen consumption during bleeding, intestinal oxygen intake is compromised due to mesenteric reflex vasoconstriction [15]. This vasoconstriction causes tissue hypoxia and continues even if the hemodynamic parameters (pulse, blood pressure, hemoglobin value, and urine output) improve due to effective fluid and blood resuscitation. Proof of tissue hypoxia is an increased lactate value and a decreased level of mixed venous oxygen saturation [16, 17]. An important place of use of the increased lactate value in the clinic is the detection of patients with secret UGI bleeding, whose pulse and hemoglobin values are normal. Therefore, we think that an increased lactate value can help to detect early gastric bleeding and support effective triage, especially in crowded emergency services. In a study, it was found that the lactate level was proportional to the increase in mortality in patients with UGI bleeding; the mean lactate level of patients was 1.95 mmol/L, while the mean level of surviving patients was 1.9 mmol/L, and the mean level of mortal patients was 4.6 mmol/L. The mortality risk of patients with lactate levels above 4 mmol/L increased 6.4 times (odds ratio) [14, 16, 17].

In a study of 154 patients admitted to the intensive care unit, lactate clearance was studied to determine the active bleeding of UGI. In this study, lactate clearance was found to be useful in detecting active bleeding [18]. In another study involving 133 patients with UGI bleeding, the mean lactate level determined in patients with and without mortality was 8.8 mmol/L and 2 mmol/L, respectively. It was determined that mortality increased as serum lactate levels increased. Lactate was found to have high sensitivity and low specificity in determining mortality [13]. In our study, as in other studies, serum lactate levels were correlated with mortality. In our study, the mean lactate level measured from venous blood was 2.63 mmol/L for all patients, 2.37 mmol/L for surviving patients and 4.80 mmol/L for mortal patients. This difference was statistically significant (*p* = 0.044). The threshold for predicting mortality was 2.32 mmol/L for lactate (66.7% sensitivity, 63.7% specificity).

The mean GBS of all patients was 11.5. The mean GBS was 11.3 in nonmortal patients and 13.3 in mortal patients. This difference was statistically significant (*p* = 0.040). GBS is a scoring system developed to predict the need for medical intervention in patients with UGI bleeding. However, in later studies, it was suggested that GBS could be used for mortality prediction, since patients who ended with mortality had higher GBS than those who were discharged [19]. In our study, GBS was found to be statistically significantly higher in patients with mortality.

Preventing unnecessary transfusions will reduce both transfusion-related complications and reduce costs. The proportion of patients undergoing blood transfusions due to UGI bleeding was 32% and 63.6% in different studies [19, 20]. In our study, red blood cell transfusion was performed in 82% of the patients. The reason for this high rate is that our hospital is the only center that can perform an endoscopy 24/7 in our city. All UGI bleeding patients are frequently referred to our hospital without transfusions. Patients who are older and have more comorbidities should be approached more sensitively when it comes to blood transfusions because of their low tolerance to anemia and tissue hypoxia. In our study, the advanced average age of patients, 75% of them having at least one comorbid disease, and the use of anticoagulant or antithrombotic drugs in 53% of the cases are considered to be other conditions increasing the frequency of transfusions. Other patients who underwent transfusions had active bleedings clinically or endoscopically, and transfusions were performed with the expectation that their hemodynamic values would decrease rapidly and their hemodynamic status would worsen. In our study, the threshold value for the predictability of erythrocyte suspension transfusion requirement was 1.58 mmol/L for lactate and 9.5 for GBS. Lactate value was found to be statistically significant but poorly correlated with predicting the need for red blood cell suspension transfusion, and GBS was statistically significant and moderately correlated. In other words, the higher the lactate value and the GBS score, the higher the need for transfusion of red blood cell. GBS is calculated according to hemoglobin value, vital signs, clinical conditions, and BUN. In UGI bleedings, the transformation of blood proteins into urea by intestinal bacteria causes its absorption from the intestine, and hypovolemia causes an increase in blood urea nitrogen (BUN) [21]. This means higher BUN and lower hemoglobin values in patients with severe hemorrhage, meaning a higher GBS. Therefore, it is thought that GBS can better estimate the need for transfusion.

The cutoff value of lactate for predicting the need for transfusion was 1.58 mmol/L. This level was lower than 2.5 mmol/L from another study conducted on patients with traumatic hemorrhagic shock [22] and 4 mmol/L from another study conducted on severe sepsis or septic shock [23]. The higher cutoff value in sepsis may be due to simultaneous bacteremia and liver damage in sepsis [24]. Traumas are often emergency situations affecting young adult patients. The mean age of the study was 38.5 years in studies where patients with traumatic hemorrhagic shock are included [22]. In our study, it is thought that the cutoff value determined for the need for transfusion is higher because of the comorbidities mentioned, the drugs used, and the lower age in these patients.

## 5. Conclusion

GBS is difficult to remember because it consists of many parameters. It also takes time to work on the Hb and BUN values. Lactate is a single biomarker that can be looked up at the bedside, where we can learn the results with the blood gas device in minutes. As a result, lactate value might be helpful in predicting the need for transfusion and predicting mortality. We think that an increased lactate value may help to detect early gastric bleeding and support effective triage, especially in crowded emergency services. Another important issue is that although lactate elevated in most severity patients admitted to the emergency department with UGI bleeding, it is still not routinely involved in any risk classification. There is a need for prospective studies in which combinations of proven risk scores and lactate are performed.

## Figures and Tables

**Table 1:** Characteristics of patients according to the requirement of transfusion.

	Transfusion group (*n* = 114)	No transfusion group (*n* = 25)	*p* value
Age (year)			
Median ± SD	64.60 ± 16.08	57.6 ± 20.33	0.630
Sex, *n* (%)			
Female	34 (29.8)	5 (20)	
Male	80 (70.2)	20 (80)	0.322
Symptoms, *n* (%)			
Melena	44 (38.6)	9 (36)	0.809
Hematemesis	35 (30.7)	10 (40)	0.368
Syncope	10 (8.8)	4 (16)	0.280
Dizziness	13 (11.4)	1 (4)	0.465
Hematochezia	8 (7.0)	1 (4)	0.494
Abdominal pain	2 (1.75)	0 (0)	0.672
Hypotension	2 (1.75)	0 (0)	0.672
Comorbidity, *n* (%)			
Heart disease	44 (38.6)	6 (24)	0.168
Hypertension	35 (30.7)	3 (12)	0.057
Diabetes mellitus	24 (21.1)	4 (16)	0.568
Renal failure	17 (14.9)	3 (12)	0.496
Cancer	18 (15.8)	1 (4)	0.197
Liver disease	10 (8.8)	1 (4)	0.689
Any other major comorbidities	11 (9.6)	2 (8)	**0.014**
No comorbidities	23 (20.2)	12 (48)	**0.004**
Medications, *n* (%)			
Antiplatelet	43 (37.7)	6 (24)	0.193
Anticoagulant	21 (18.4)	4 (16)	0.517
NSAID	16 (14)	6 (24)	0.232
No medications	32 (28)	12 (48)	0.086
Endoscopic findings, *n* (%)			
Duodenal ulcer	47 (41.2)	9 (36)	0.629
Gastric ulcer	27 (23.7)	7 (28)	0.649
Esophageal variceal bleeding	12 (10.5)	3 (12)	0.734
Esophageal ulcer/erosive esophagitis	11 (9.6)	2 (8)	0.575
Erosive gastritis	9 (7.9)	3 (12)	0.452
Esophageal cancer	8 (7)	0 (0)	0.350
Gastric cancer	0 (0)	1 (4)	0.180
Endoscopic hemostasis, *n* (%)			
Sclerotherapy+heater probe	34 (29.8)	3 (12)	0.068
Sclerotherapy	14 (12.2)	4 (16)	0.742
Band ligation	7 (6.1)	2 (8)	0.664
Sclerotherapy+argon plasma coagulation	7 (6.1)	1 (4)	0.561
Sclerotherapy+hemoclip	7 (6.1)	0 (0)	0.351
Sclerotherapy+band ligation	3 (2.6)	0 (0)	0.549
Argon plasma coagulation	3 (2.6)	0 (0)	0.549
No endoscopic hemostasis	39 (34.2)	15 (60)	**0.017**
Rebleeding, *n* (%)	23 (20.2)	2 (8)	0.248
Surgery need, *n* (%)	4 (3.5)	0 (0)	0.448
Prognosis			
Survival	99 (86.8)	25 (100)	0.073
Nonsurvival	15 (13.2)	0 (0)	
Hypotension (SBP < 90 mmHg), *n* (%)	29 (25.4)	1 (4)	**0.007**
Tachycardia (pulse > 100 beats/min), *n* (%)	54 (47.4)	10 (40)	0.813
Hemoglobin value (g/dL), median ± SD	8.4 ± 2.24	12.06 ± 1.98	<**0.001**
Blood urea nitrogen value (mg/dL), median ± SD	55.8 ± 30.6	36.2 ± 19.2	**0.003**
Venous lactate value (mmol/L), median ± SD	2.86 ± 2.49	1.58 ± 0.68	<**0.001**
Glasgow-Blatchford Score value, median ± SD	12.4 ± 3.05	7.12 ± 2.7	<**0.001**

**Table 2:** Characteristics of patients according to survival.

	Survival group (*n* = 124)	Nonsurvival group (*n* = 15)	*p* value
Age (year)			
Median ± SD	62.7 ± 17.28	68 ± 14.8	0.264
Sex, *n* (%)			
Female	34 (27.4)	5 (33.3)	
Male	90 (72.6)	10 (66.6)	0.761
Symptoms, *n* (%)			
Melena	50 (40.3)	3 (20)	0.126
Hematemesis	36 (29)	9 (60)	**0.021**
Syncope	14 (11.3)	0 (0)	0.363
Dizziness	13 (10.5)	1 (6.6)	0.537
Hematochezia	8 (6.5)	1 (6.6)	0.654
Abdominal pain	1 (0.8)	1 (6.6)	0.205
Hypotension	2 (1.6)	0 (0)	0.795
Comorbidity, *n* (%)			
Heart disease	47 (37.9)	3 (20)	0.172
Hypertension	33 (26.6)	5 (33.3)	0.554
Diabetes mellitus	25 (20.2)	3 (20)	0.646
Renal failure	17 (13.7)	3 (20)	0.454
Cancer	12 (9.6)	7 (46.6)	**0.001**
Liver disease Any other major comorbidities	8 (6.5) 12 (9.7)	3 (20) 1 (6.6)	0.099 0.168
No comorbidities	34 (27.4)	1 (6.6)	0.115
Medications, *n* (%)			
Antiplatelet	46 (37.1)	3 (20)	0.191
Anticoagulant	22 (17.7)	3 (20)	0.734
NSAID	20 (16.1)	2 (13.3)	0.565
No medications	37 (29.8)	7 (46.6)	0.394
Endoscopic findings, *n* (%)			
Duodenal ulcer	51 (41.1)	5 (33.3)	0.561
Gastric ulcer	32 (25.8)	2 (13.3)	0.360
Esophageal variceal bleeding	15 (12.1)	0 (0)	0.371
Esophageal ulcer/erosive esophagitis	10 (8.1)	3 (20)	0.150
Erosive gastritis	11 (8.9)	1 (6.6)	0.620
Esophageal cancer	4 (3.2)	4 (26.6)	**0.005**
Gastric cancer	1 (0.8)	0 (0)	0.892
Endoscopic hemostasis, *n* (%)			
Sclerotherapy+heater probe	33 (26.6)	4 (26.6)	0.605
Sclerotherapy	18 (14.5)	0 (0)	0.218
Band ligation	8 (6.5)	1 (6.6)	0.654
Sclerotherapy+argon plasma coagulation	7 (5.6)	1 (6.6)	0.609
Sclerotherapy+hemoclip	7 (5.6)	0 (0)	0.441
Sclerotherapy+band ligation	2 (1.6)	1 (6.6)	0.292
Argon plasma coagulation	1 (0.8)	2 (13.3)	0.031
No endoscopic hemostasis	48 (38.7)	6 (40)	0.923
Rebleeding, *n* (%)	22 (17.7)	3 (20)	0.734
Surgery need, *n* (%)	3 (2.4)	1 (6.6)	0.370
Hypotension (SBP < 90 mmHg), *n* (%)	23 (18.5)	7 (46.6)	0.104
Tachycardia (pulse > 100 beats/min), *n* (%)	53 (42.7)	11 (73.3)	0.445
Hemoglobin value (g/dL), median ± SD	9.1 ± 2.62	9.1 ± 2.46	0.961
Blood urea nitrogen value (mg/dL), median ± SD	47.9 ± 23.5	88.3 ± 48.6	**0.006**
Venous lactate value (mmol/L), median ± SD	2.37 ± 1.8	4.8 ± 4.2	**0.044**
Glasgow-Blatchford Score value, median ± SD	11.3 ± 3.6	13.3 ± 3.4	**0.040**

**Table 3:** ROC analysis of lactate values and GBS for need for red blood cell transfusion.

	AUC	SD	95% CI	Cutoff	Sensitivity	Specificity	*p*
GBS	0.904	0.029	0.848-0.961	9.5	83.3	80	<0.001
Lactate	0.689	0.050	0.590-0.788	1.58	70.2	60	0.003

AUC: area under the curve; CI: confidence interval; GBS: Glasgow-Blatchford Bleeding Score; SD: standard deviation.

**Table 4:** ROC analysis of lactate values and GBS for hospital mortality.

	AUC	SD	95% CI	Cutoff	Sensitivity	Specificity	*p*
GBS	0.683	0.078	0.530-0.835	13.5	66.7	71	0.021
Lactate	0.664	0.086	0.495-0.834	2.32	66.7	63.7	0.038

AUC: area under the curve; SD: standard deviation; CI: confidence interval; GBS: Glasgow-Blatchford Bleeding Score.

## Data Availability

The study protocol, statistical analysis plan, informed consent form, analytic code, and main manuscript data used to support the findings of this study were supplied by Muge Gulen under license and so cannot be made freely available. Requests for the access to these data should be made by contacting Muge Gulen, email address: muge-gulen@hotmail.com.
